# Single-cell RNA sequencing of neurofibromas reveals a tumor microenvironment favorable for neural regeneration and immune suppression in a neurofibromatosis type 1 porcine model

**DOI:** 10.3389/fonc.2023.1253659

**Published:** 2023-09-25

**Authors:** Dalton T. McLean, Jennifer J. Meudt, Loren D. Lopez Rivera, Dominic T. Schomberg, Derek M. Pavelec, Tyler T. Duellman, Darya G. Buehler, Patrick B. Schwartz, Melissa Graham, Laura M. Lee, Keri D. Graff, Jamie L. Reichert, Sandra S. Bon-Durant, Charles M. Konsitzke, Sean M. Ronnekleiv-Kelly, Dhanansayan Shanmuganayagam, C. Dustin Rubinstein

**Affiliations:** ^1^ Biotechnology Center, University of Wisconsin–Madison, Madison, WI, United States; ^2^ Molecular & Environmental Toxicology Program, University of Wisconsin–Madison, Madison, WI, United States; ^3^ Biomedical & Genomic Research Group, Department of Animal and Dairy Sciences, University of Wisconsin–Madison, Madison, WI, United States; ^4^ Department of Pathology and Laboratory Medicine, University of Wisconsin School of Medicine and Public Health, Madison, WI, United States; ^5^ Department of Surgery, University of Wisconsin School of Medicine and Public Health, Madison, WI, United States; ^6^ Research Animal Resources and Compliance (RARC), Office of the Vice Chancellor for Research and Graduate Education, University of Wisconsin–Madison, Madison, WI, United States; ^7^ Swine Research and Teaching Center, Department of Animal and Dairy Sciences, University of Wisconsin–Madison, Madison, WI, United States; ^8^ Center for Biomedical Swine Research and Innovation, University of Wisconsin–Madison, Madison, WI, United States

**Keywords:** neurofibromatosis type 1 (NF1), single cell RNA seq, swine, tumor microenvironment (TME), neurofibroma, cancer-associated fibroblasts (CAF), immune checkpoint, neuroregeneration

## Abstract

Neurofibromatosis Type 1 (NF1) is one of the most common genetically inherited disorders that affects 1 in 3000 children annually. Clinical manifestations vary widely but nearly always include the development of cutaneous, plexiform and diffuse neurofibromas that are managed over many years. Recent single-cell transcriptomics profiling efforts of neurofibromas have begun to reveal cell signaling processes. However, the cell signaling networks in mature, non-cutaneous neurofibromas remain unexplored. Here, we present insights into the cellular composition and signaling within mature neurofibromas, contrasting with normal adjacent tissue, in a porcine model of NF1 using single-cell RNA sequencing (scRNA-seq) analysis and histopathological characterization. These neurofibromas exhibited classic diffuse-type histologic morphology and expected patterns of S100, SOX10, GFAP, and CD34 immunohistochemistry. The porcine mature neurofibromas closely resemble human neurofibromas histologically and contain all known cellular components of their human counterparts. The scRNA-seq confirmed the presence of all expected cell types within these neurofibromas and identified novel populations of fibroblasts and immune cells, which may contribute to the tumor microenvironment by suppressing inflammation, promoting M2 macrophage polarization, increasing fibrosis, and driving the proliferation of Schwann cells. Notably, we identified tumor-associated *IDO1* ^+^/CD274^+^ (*PD-L1)* ^+^ dendritic cells, which represent the first such observation in any NF1 animal model and suggest the role of the upregulation of immune checkpoints in mature neurofibromas. Finally, we observed that cell types in the tumor microenvironment are poised to promote immune evasion, extracellular matrix reconstruction, and nerve regeneration.

## Introduction

Neurofibromatosis Type 1 (NF1) is a complex monogenic disorder that affects 1 in 3000 children annually, making it one of the most common genetically inherited disorders (1); it results from mutations of the neurofibromin 1 (*NF1*) gene. The complexity of NF1 is largely due to the variability in mutations. Over 3,000 different germline *NF1* mutations have been identified with varying and poorly understood genotype–phenotype relationships (2). Individuals with NF1 are prone to developing benign and malignant peripheral nervous system tumors (e.g., neurofibromas, malignant peripheral nerve sheath tumors) and central nervous system tumors (e.g., optic pathway glioma, malignant glioma). Throughout their lifetime, 99% of NF1 patients will develop superficial cutaneous neurofibromas, and up to 50% will develop plexiform neurofibromas (pNFs) (3). Patients with pNFs experience an 8-13% lifetime risk of progression to highly aggressive malignant peripheral nerve sheath tumors (MPNSTs) (4–6).

Neurofibromas arise as a bulbous expansion of peripheral nerve fascicles and contain a neoplastic Schwann cell population along with remaining sensory axons, myelinating-Schwann cells, endoneurial fibroblasts, vascular smooth muscle cells, endothelial cells, and various immune cells (7). In addition to this cellular composition, other hallmarks of neurofibromas include modified collagenous matrices, over-expression of growth factors, and the presence of additional types of fibroblasts (perineurial/epineurial) (8). NF1 tumorigenesis relies upon the somatic loss of heterozygosity of *NF1* in the Schwann cell lineage and support from *NF1* haploinsufficiency in other cell types in the surrounding microenvironment (9–13). However, migrating neural crest cells, often the target for *NF1* manipulation in rodent models, develop into two early-stage cell types, Schwann cell progenitors and immature Schwann cells (14). These two cell types can then differentiate into melanocytes, endoneurial fibroblasts, parasympathetic neurons, myelinating Schwann cells, and non-myelinating Schwann cells (15).

Genetically engineered mouse models have effectively pointed towards a Schwann cell lineage cell of origin for pNFs (16). However, these mouse models do not fully recapitulate the disease spectrum seen in NF1 patients. For example, more complex mouse models employing the Cre-Lox system to generate a bi-allelic loss of *NF1* in a specific cell lineage (e.g., Schwann cell) often require mutations in other tumor suppressor genes (such as *TP53* or *INK4a/ARF* that are frequently mutated in NF1-associated tumors) to successfully generate neurofibromas (17, 18), albeit with major limitations. In mouse models designed to study advanced neurofibromas, MPNSTs often develop rapidly and asynchronously *de novo* rather than arising from established pNFs, as observed in humans (19). Consequently, preclinical studies in these mice often have not been predictive of drug efficacy in humans (20, 21).

Porcine models benefit from genetic and physiologic similarities to humans that include chromosomal synteny, aging rate, organ size, and body size. Two previously developed NF1 pig models (22, 23), along with the three porcine models with unique mutations developed by us using more advanced molecular validation methods (24), display many of the hallmarks of the disorder in humans. Furthermore, because the biological clock of pigs, derived from epigenetic profiles of mapped and conserved CpG islands, closely matches that of a human (25), it is likely that spontaneous neurofibromas in pigs will grow and progress over a much longer window than can be observed in mice, which can quickly succumb to a rapidly growing tumor. We hypothesized that porcine models would be suitable for dissecting long-term cell signaling and transformation present in mature pNFs. The spontaneous formation of tumors in our NF1 pigs and our infrastructural capabilities for maintaining porcine models for long study periods provided the appropriate research opportunity. In this paper, we validated the development of spontaneous neurofibromas in our porcine NF1 model, which grew over two years of the animal’s life, and coupled this with a comprehensive single-cell RNA sequencing (scRNA-seq) study of the spontaneous neurofibromas and adjacent normal tissue.

## Materials and methods

### Porcine tumor and non-tumor tissue collection for scRNA-seq

Experiments involving animals were conducted under protocols approved by the University of Wisconsin–Madison Institutional Animal Care and Use Committee in accordance with published National Institutes of Health and United States Department of Agriculture guidelines. We have previously published on the creation and genomic validation of our three distinct NF1 porcine models, including the one utilized in this study (24). The present study used tissue samples (two tumors and an adjacent normal region) collected from a 3.83-year-old male NF1 pig harboring an *NF1* exon a31 excision producing an alternatively spliced *NF1* allele, whose regulation is associated with disease severity (24, 26). Development of the masses was first noted when the animal was about 11 months of age; the masses increased in size with age until the animal was euthanized for necropsy and tissue collection. The pig was bred and housed at the UW Swine Research and Teaching Center, a closed-herd, specific-pathogen-free (SPF) facility. The pig was transported to an on-campus facility on the day of the necropsy. The pig was initially sedated with intramuscular administration of a cocktail of Telazol^®^ (3.3 mg/kg; Zoetis Inc., Kalamazoo, MI, USA), xylazine (1.6 mg/kg; Bimeda, Inc., Oakbrook Terrace, IL, USA), and ketamine (1.6 mg/kg; Zoetis Inc.). An additional dose of ketamine (2 mg/kg) was given intramuscularly before euthanasia with intracardiac administration of Fatal-Plus^®^ Euthanasia Solution (Vortech Pharmaceutical Ltd, Dearborn, MI, USA) according to the manufacturer’s recommendations. The necropsy was led by two board-certified veterinary pathologists.

**Figure 1:**
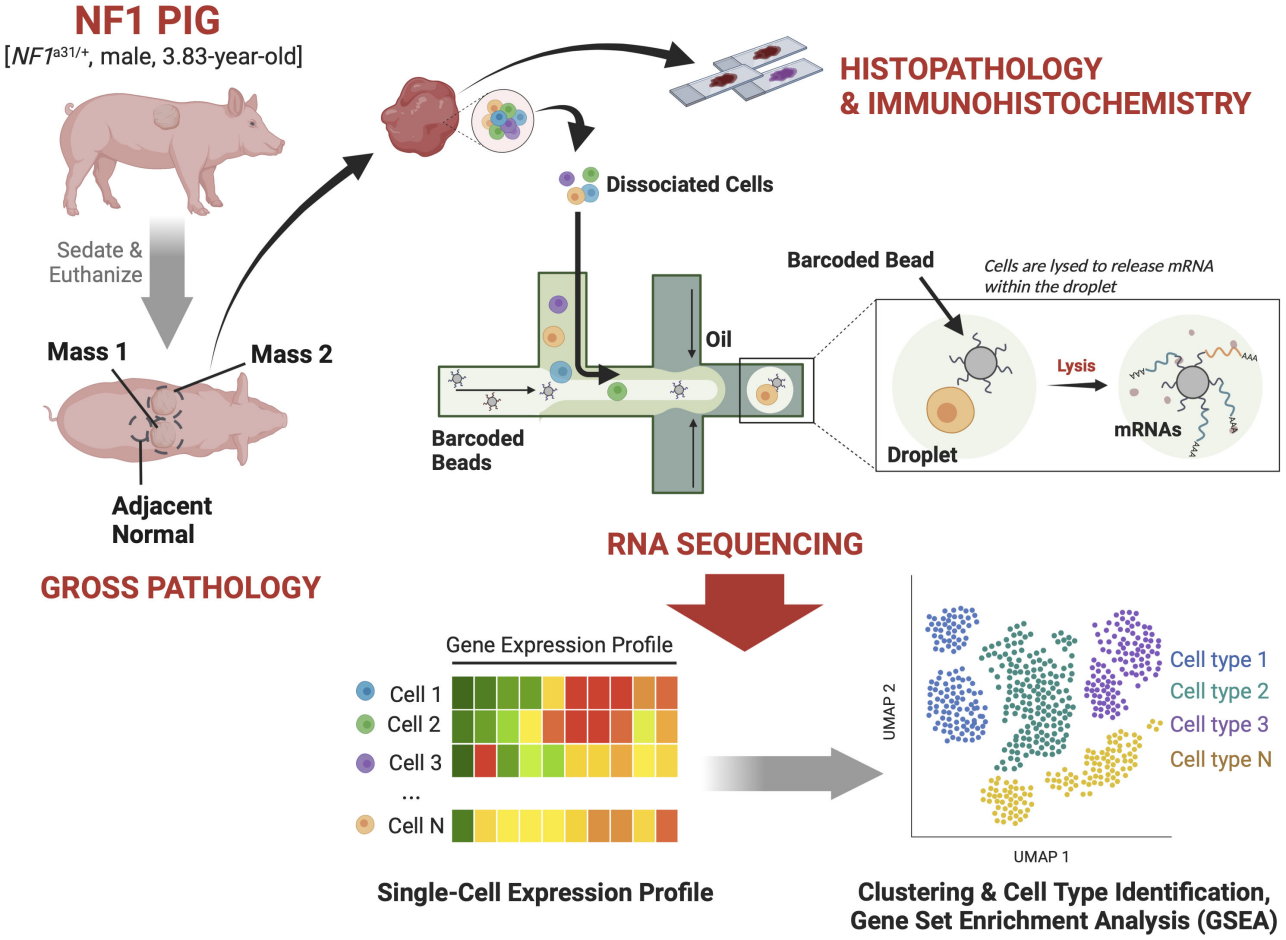
Study Design: A 3.83-year-old male NF1 pig harboring an excision in exon 31 of *NF1* and two caudodorsal masses of interest was sedated and then euthanized. The masses and an adjacent normal region were resected out and sectioned for histopathological validation and scRNA-seq analysis.

Caudodorsal subcutaneous masses were identified on the right and left sides of the thorax and resected, providing two samples we have called Mass 1 and Mass 2 (**Figure 1**). A non-tumor, normal adjacent sample was collected to serve as the control. Upon gross examination, the masses were primarily located in the subcutaneous tissue and extended into the overlying dermis. Like human neurofibromas, both masses appeared light-yellow to tan in color, semi-firm, and variably demarcated from surrounding tissue. Mass 1 was located on the right caudodorsal thorax, 88 cm caudal to the base of the right pinna, and 12 cm to the right of midline, while Mass 2 was located left caudodorsal thorax, at approximately the same level as the right caudodorsal mass, 10.5 cm left of midline. Once incised around the masses, Mass 1 was approximately 6 x 5 x 3.2 cm, while Mass 2 was 11.7 x 11.7 x 3.2 cm. Each tumor tissue sample was taken from the center of the mass and divided into two segments for histological and immunohistochemical validation and the scRNA-seq analysis.

### Histology and immunohistochemistry

Tissue sections were processed and embedded using an adapted protocol (Abcam, Cambridge, UK) (27). Briefly, following fixation with 10% neutral buffered formalin, tissues placed in processing/embedding cassettes were dehydrated using increasing ethanol concentrations. This was followed by clearing with three changes of xylene for 20 minutes each. Xylene was then exchanged with two paraffin changes (60 minutes each) to allow adequate tissue infiltration of the wax and then embedded. The embedded tissues were cut into 5 µm sections for the conventional hematoxylin and eosin (H&E) and IHC staining. The IHC protocol was adapted from R&D Systems (Minneapolis, MN, USA) (28). Heat-induced antigen retrieval was performed using a citrate buffer at pH 7.4 (29). The following antibodies were used to classify the tumors: S100 (1:4000; Agilent Dako, Santa Clara, CA, USA; GA50461-2), CD34 (1:400; Bioss, Woburn, MA, USA; bs-8996R), Sox10 (1:100; Abcam, Cambridge, UK; ab227680), CD117 (1:100, Cell Signaling; 37805S), GFAP (1:200, Abcam; ab16997), AIF1 (1:500, LSBio; LS-B2645), and SMA (1:250, LSBio; LS-B3933). Briefly, the primary antibodies were incubated overnight at 4°C while the secondary antibodies, including anti-rabbit, anti-goat, and anti-mouse (1:250, Abcam; ab6728, ab6885, ab6721, respectively), were incubated on slides for 30 minutes at room temperature. The targets of interest were detected using a DAB chromogen substrate kit (Cell Signaling; #8059) and counterstained using Mayer’s Hematoxylin for 30 seconds (Abcam; ab220365).

### RNA extraction and single-cell sequencing

The samples for the scRNA-seq analysis were promptly placed into 1.5 mL conical tubes on ice until enzymatically digestion could be started within the hour to obtain a single cell suspension (30). Briefly, each tissue sample was minced into <4 mm pieces and digested with a Tumor Dissociation Kit (Cat #130-095-929; Miltenyi Biotec, Gaithersburg, MD, USA) according to the manufacturer’s instructions. The resulting suspensions were further digested using collagenase II to obtain sufficient cell counts for scRNA-seq (>50,000 cells/sample) (31). Libraries were constructed according to the Chromium Single Cell 3’ Reagent Kit v3.1 user guide (10x Genomics, Pleasanton, CA) by the UW Gene Expression Center. Briefly, cell concentration and cell viability of the single-cell suspension were quantified on the Countess II (Thermo Fisher Scientific, Waltham, MA) using 0.4% Trypan Blue (Invitrogen, Carlsbad, CA). The appropriate volume of cells was loaded onto the Chromium Single Cell Chip G (10X) required for yielding a cell recovery of approximately 50,000 cells. After completing the Chromium run, the gel beads-in-emulsion (GEMs) were transferred to emulsion-safe strip tubes for GEM-RT using an Eppendorf MasterCycler Pro thermocycler (Eppendorf, Hamburg, Germany). Following RT, GEMs were broken, and the pooled single-cell cDNA was amplified. Post-cDNA amplified product was purified using SPRIselect (Beckman Coulter, Brea, CA) and quantified on a Bioanalyzer 2100 (Agilent, Santa Clara, CA) using the High Sensitivity DNA kit (Agilent, Santa Clara, CA). Full-length cDNA was fragmented and used to generate cDNA libraries according to the standard 10X Genomics workflow. These libraries were sequenced on a NovaSeq 6000 Sequencing System (Illumina Inc., San Diego, CA) with paired-end 150bp sequencing by the UW DNA Sequencing Facility.

### Preprocessing, mapping, and alignment

The scRNA-Seq data were analyzed by the UW Bioinformatics Resource Center. Experiment data were demultiplexed using the Cell Ranger Single Cell Software Suite and aligned to Sscrofa11.1, Barcode counting, UMI counting, and gene expression estimation for each sample according to the 10x Genomics documentation (32). The gene expression estimates from each sample were then aggregated using Cellranger (cellranger aggr) to compare experimental groups with normalized sequencing depth and expression data.

### Quality control, data integration, visualization, and clustering

Analysis was performed with R Statistical Software (v4.1.3) and Seurat (v4.2.0) (33). Each sample (Mass 1, Mass 2, and Normal Adjacent Tissue) was individually processed for quality control before integration. Seurat objects were created with thresholds: min.cells = 3, min.features = 200. We used ENSEMBL to identify human orthologs of porcine genes, and manually used VGNC to identify remaining orthologs. Next, data was subsetted based on a threshold for 18 mitochondrial genes set at <10% representation and <1% representation of Hemoglobin Beta (**Supplementary Table 1**). The threshold for unique features per cell was >200 and <6000 to avoid low-quality cells and doublets. Seurat objects for each dataset were merged then split to create lists that can be transformed and normalized using *SCTransform* (34).

### Cell type identification

Initial clustering divided the cell population into 14 individual clusters, which were automatically identified with *ScType* (**Supplementary Table 2**) using a modified marker list generated by converting human gene symbols to orthologous *Sus scrofa* gene symbols (35). Lists of cell types were curated and included markers for all possible cell types within these tissues, and subsequent cell type scores were generated using the full scRNA-seq dataset and differentially expressed genes (DEGs) (35). Automatically scored and identified cell types were then verified manually using commonly known cell type markers (**Supplementary Table 1**). Violin plots for cell types are depicted using the raw RNA counts for genes of interest.

### Differentially expressed genes

Analysis of gene expression was performed using Seurat v4.2.0 (**Supplementary Document**), and DEGs were identified using the normalized RNA counts, or the SCT assay. To prepare the Seurat object to run differential expression testing on the SCT assay, we used *PrepSCTFindMarkers().* For bulk comparisons of tumor vs. normal, we used *RenameIdent()* to merge both Mass 1 and Mass 2. Populations were subsetted for comparison using *Subset().* Pairwise comparisons between clusters was made using *FindMarkers()* and specific identification of clusters. DEGs in one cluster compared to all other clusters were done using *FindAllMarkers().* The default Wilcoxon Rank Sum test was used for analysis. DEGs were defined as being expressed in >20% of the cluster, with |logFC| >0.25, and adjusted p-value <0.05 (**Supplementary Tables 3**, **4**).

### Gene set enrichment analysis and gene ontology

DEG lists were populated with *Sus scrofa* Ensembl IDs. First, we automatically converted Ensembl IDs using “biomaRt” datasets “sscrofa_gene_ensembl” and “hsapiens_gene_ensembl”. This method was only successful in converting ~70% of *Sus scrofa* Ensembl IDs to human gene symbols. The remaining IDs were then converted manually, and the list was uploaded to R to be used for GSEA. The Fast Gene Set Enrichment Analysis (fgsea) package was used to analyze enriched pathways from the GO: Gene Ontology gene sets (36). The top 15 enriched pathways were visualized by adjusted p-value initially, and only pathways with an adjusted p-value <0.05 were used for further analysis. Enrichment plots were visualized using *plotEnrichment()* of specified pathways (**Supplementary Table 5**).

## Results and Discussion

### Histological validation of porcine neurofibromas

**Figure 2:**
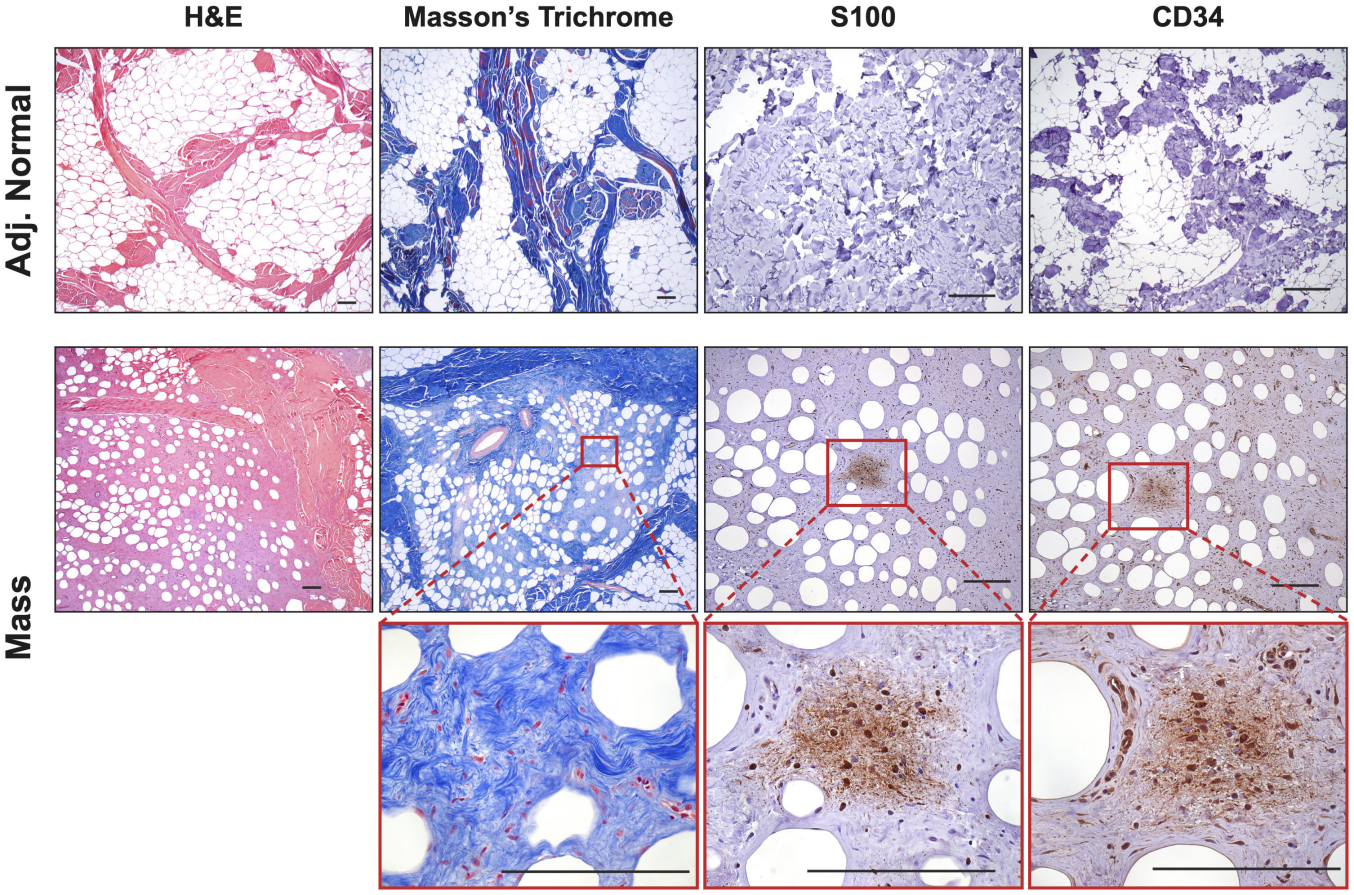
Histological Confirmation of Neurofibromas: Representative samples (Mass 1) were stained with H&E and Masson’s Trichrome to examine tumor architecture and collagen deposition. Samples contained mixed regions of high and low cellularity, heavy collagen deposition, spindle-shaped cells, interspersed immune cells, small axons, and blood vessels. Masson’s Trichrome revealed intricate collagen deposition, including large bands surrounding areas of neurofibroma and small wire-like fibrils within. The tumors contained areas that were S100^+^ and CD34^+^, particularly in areas of hypercellularity. Scale bars: 200 µm.

**Figure 3:**
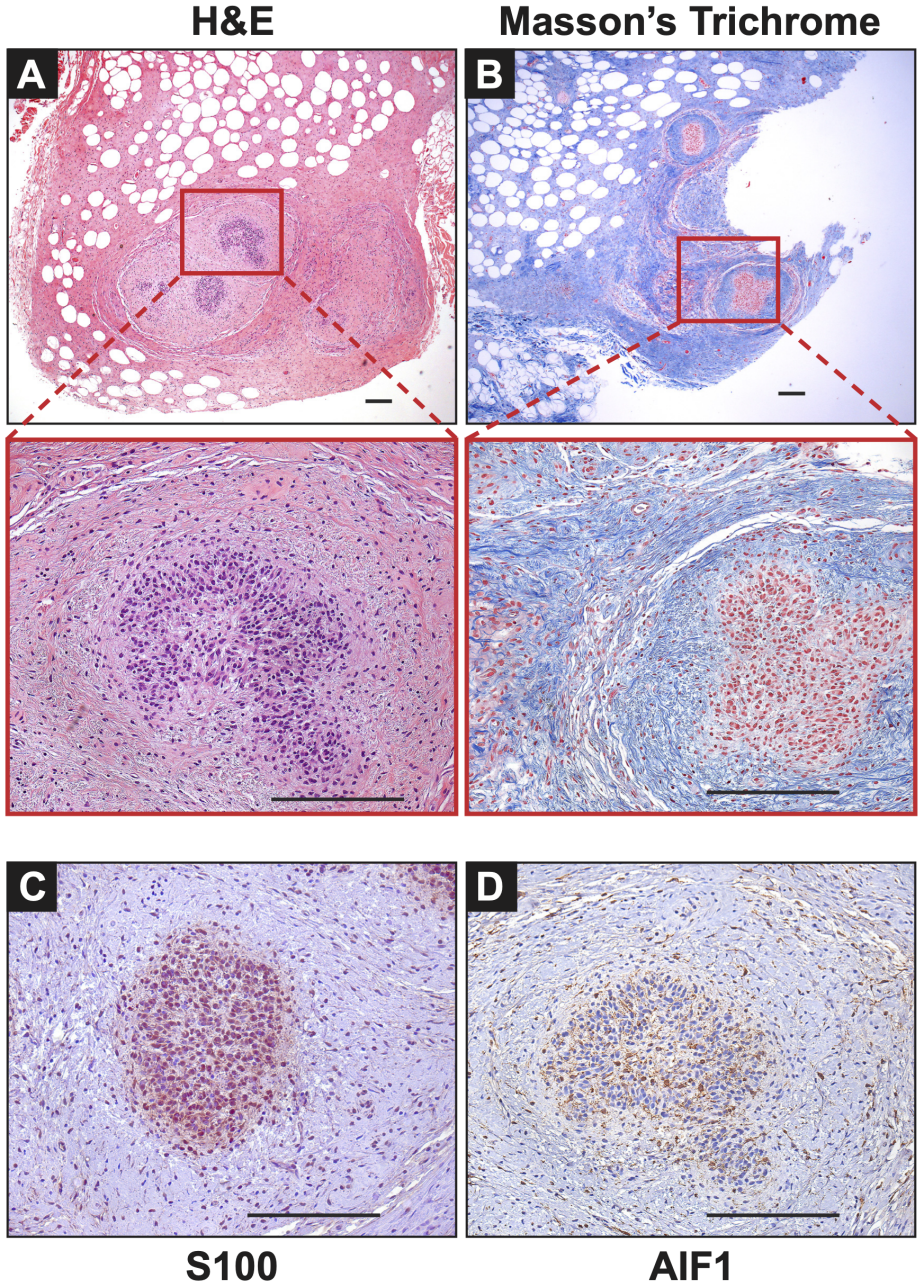
Notable Features of Neurofibromas: **(A, B)** In Mass 2, multiple nodules of proliferating Schwann cells were observed, surrounded by concentric rings of collagen. **(C)** These bundles stained positive for S100. **(D)** AIF1 immunostaining reveals the presence of infiltrating macrophages alongside Schwann cells. Scale bars: 200 µm.

Extensive phenotyping of the focal study animal (and over 30 others from our three NF1 porcine models) will be published elsewhere. Here, we focus on the validation of the two resected masses. The tissues were first stained with hematoxylin and eosin (H&E) and Masson’s Trichrome to examine tumor architecture and collagen deposition. Both masses demonstrated typical histologic features of diffuse neurofibroma involving subcutaneous adipose tissue and showing an admixture of Schwann cells with wavy, slightly hyperchromatic nuclei, admixed with fibroblasts and fibroblast-like cells, small axons, interspersed immune cells, thick or wavy collagen bundles, and blood vessels (**Figure 2**). The presence of lesional collagen was highlighted by Masson’s Trichrome staining (**Figures 2**, **3**). Hypercellular Schwann cell-rich zones, Verocay bodies, and nuclear palisading indicative of schwannomas were not observed (37). The adjacent normal tissue was primarily composed of dense collagen bundles surrounding areas of adipose. Mononuclear, interspersed immune cells morphologically resembling mast cells were present in low abundance, but we could not objectively confirm this cell type due to the absence of a compatible cKIT antibody. Mass 2 showed similar findings of diffuse neurofibroma; but some areas also showed S100^+^ Schwann cell-rich nodules surrounded by concentric rings of collagen, immune cells, and other cell types (**Figure 3**). Neither case showed convincing precursor plexiform neurofibroma or could be traced to the originating nerve. Therefore, the overall morphologic findings strongly favored diffuse neurofibromas, most likely of plexiform origin (diffuse plexiform neurofibromas, dpNFs) rather than of cutaneous origin.

Immunohistochemistry revealed that both tumors displayed strong cytoplasmic and nuclear positivity for two proteins used in neurofibroma diagnosis, S100 positivity in Schwann cells and CD34, which highlighted fibroblasts, fibroblast-like cells, and a subset of Schwann cells (38). The adjacent normal tissue showed little to no positivity for these markers (**Figure 2**). Tumors also focally stained positive for neural cell origin markers GFAP and SOX10, particularly in areas of hypercellularity (data not shown) (38). Immunostaining for smooth muscle actin (SMA), a myofibroblastic and smooth muscle marker, produced little to no positivity except for a few larger vessels that can also be visualized by red Masson’s Trichrome staining (**Figure 2**) (39, 40). To summarize, we observed the typical histologic components of neurofibromas, including mixed cellularity, spindle-shaped Schwann cells, neurofibroma-associated fibroblasts, coarse and wire-like collagen bundles, blood vessels and moderate immune infiltration, including mononuclear cells histologically similar to mast cells. The lesional spindle cells were S100^+^, SOX10^+^, GFAP^+^, and CD34^+^, in keeping with known human neurofibroma markers. With this data, we can conclude that the two masses (Mass 1 and Mass 2) accurately recapitulate the histology of neurofibromas from human NF1 patients by being positive for necessary neurofibroma markers and reproducing many of the essential features common to neurofibromas.

### Single cell analysis of swine neurofibromas

**Figure 4:**
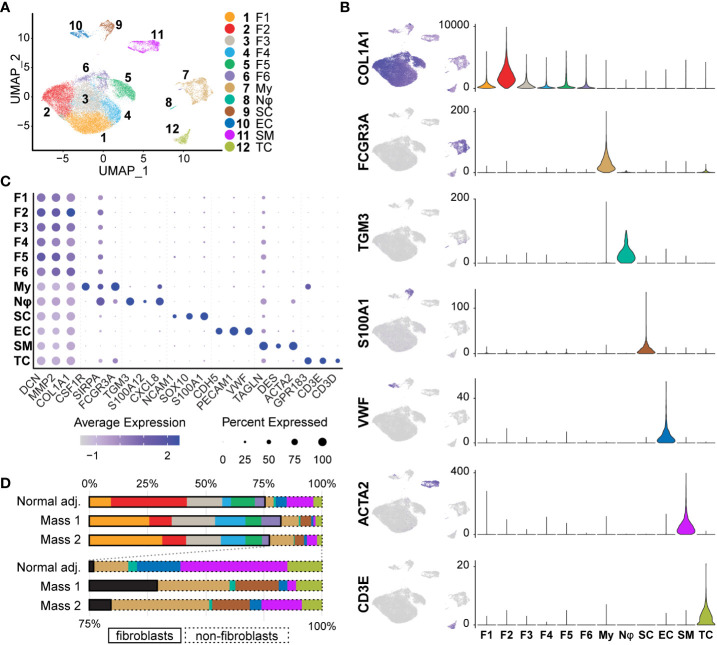
Identification of Tissue Cell Types: **(A)** Initial UMAP separated cell types into 14 clusters that were then merged into 12 clusters of 7 distinct cell classes. **(B)** Cell types were identified using a manual selection of cell type markers and automatic identification based on scores generated by *ScType()*. Feature maps (relative expression) and violin plots (RNA counts; y-axis) depict specificity for a select marker for each cell type. Fibroblasts (*COL1A1^+^*), Myeloid cells (*FCGR3A^+^*), Neutrophils (*TGM3^+^*), Schwann cells (*S100A1^+^*), Endothelial (*VWF^+^*), Smooth muscle/pericytes (*ACTA2^+^*), and T cells (*CD3E^+^*), **(C)** Three markers were used to verify each cell type manually. The relative expression of each is depicted in the dot plot. Fibroblast clusters share higher relative expression of *COL1A1, MMP2*, and *DCN*. Dot size represents the percentage of cells with expression of the marker and color intensity depicts the average expression level. **(D)**The proportions of most cell types were similar across samples. Schwann cells were mostly derived from tumor samples (98.4%). Myeloid cells were enriched in tumor samples (9.30% vs. 3.84%). Fibroblasts represented over 70% of all samples. For this and all future figures, the naming convention is as follows: Fibroblast 1 (F1), Fibroblast 2 (F2), Fibroblast 3 (F3), Fibroblast 4 (F4), Fibroblast 5 (F5), Fibroblast 6 (F6), Myeloid Cells (My), Neutrophils (Nφ), Schwann cells (SC), Endothelial cells (EC), Smooth Muscle/Pericytes (SM), T Cells (TC).

After single-cell dissociation, library preparation, sequencing, data integration and normalization, we were left with 31,211 individual cell transcriptomes with an average of 2,980 unique genes per cell. To then identify shared clusters and associated cell types, all samples were subject to dimensional reduction and non-supervised data clustering as described in *Materials and Methods*. The UMAP (Uniform Manifold Approximation and Projection) from this analysis initially outputted 14 distinct clusters of cells that were later combined into 12 clusters (**Figure 4A**). Using both automatic and manual cell typing, we identified Schwann cells (*S100A1^+^, SOX10^+^, NCAM1^+^*), fibroblasts (*COL1A1^+^, MMP2^+^, DCN^+^*), myeloid cells (*FCGR3A^+^, CSF1R^+^, SIRPA^+^*), vascular smooth muscle cells/pericytes (*ACTA2^+^, TAGLN^+^, DES^+^*), endothelial cells (*vWF^+^, PECAM1^+^, CDH5^+^*), T cells (*GPR183^+^*, *CD3D^+^, CD3E^+^*), and neutrophils (*TGM3^+^, CXCL8^+^, S100A12^+^*) (**Figures 4B**, **4C**). Histological analysis of human neurofibromas and those from mouse models have established that the cells of origin of neurofibromas are from the Schwann cell lineage and that supporting cells within the tumors include fibroblasts, vascular smooth muscle cells, immune cells, and endothelial cells (11, 22, 41, 42). Schwann cells were found almost exclusively in our tumor samples when compared to the normal adjacent sample and made up 4.37% and 0.14% of all cells, respectively (**Figure 4D**). Because this analysis and further analyses did not identify noticeable differences between Mass 1 and Mass 2 sample data, data from these samples were pooled for further analysis. We did not specifically identify perineurial or endoneurial fibroblasts, but we suspect these cell types are among one or more fibroblast clusters.

### M2 macrophages and dendritic cells promote an immunosuppressive microenvironment

**Figure 5:**
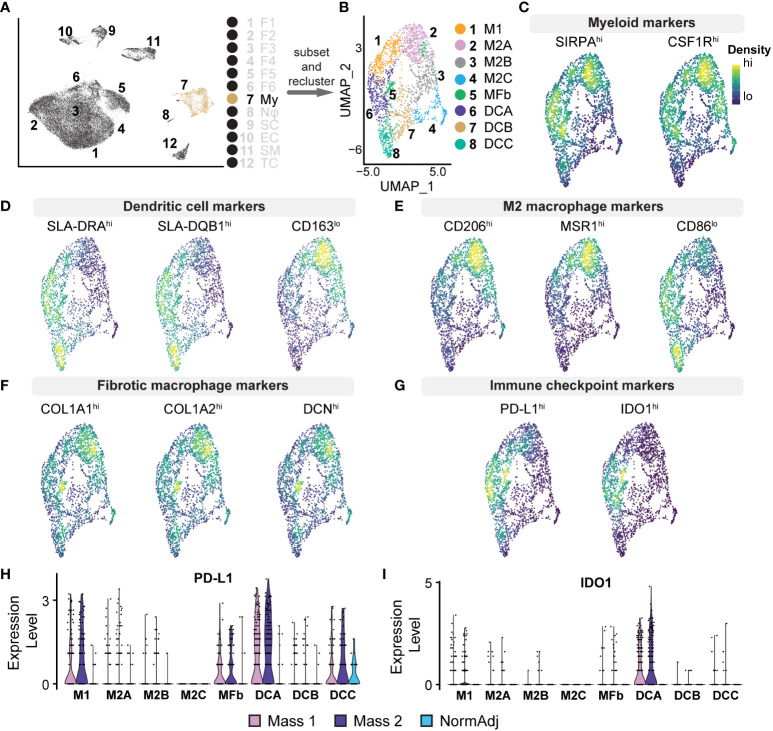
Analysis of Myeloid Cell Populations: **(A)** Cluster 7 was subsetted from the full dataset for subsequent analysis. **(B)** UMAP analysis on myeloid cell subset produced 7 unique clusters. **(C–G)** Density plots highlighting gene expression intensity scaled from high (“hi”) to low (“lo”) of selected subtype markers. Subscripts adjacent to markers reflect the expected phenotype for the above cell type. **(C)** Pan-myeloid markers *SIRPA^+^* and *CSF1R^+^* were used for initial cluster identification. **(D)** Specificity of subtypes was determined by the ratio of expression of the following factors: Dendritic cells (*SLA-DRA^hi^, SLA-DQB1^hi^, CD163^lo^*). **(E)** M2 macrophages (*CD206^hi^, MSR1^hi^, CD86^lo^*) **(F)** Fibrotic macrophages (*COL1A1^hi^, COL1A2^hi^, DCN^hi^*). **(G)** Density plots for *PD-L1* (*CD274*) and *IDO1* reveal positivity in dendritic cells, M1 macrophages, and fibrotic macrophages. **(H)** Relative expression violin plots reveal *PD-L1* was predominantly expressed in tumorigenic dendritic cells and M1 macrophages with a small population of *PD-L1^+^* dendritic cells found in adjacent normal tissue. **(I)** Immune checkpoint *IDO1* was found predominantly in dendritic cells and exclusively found in tumors.

The tumor microenvironment was enriched for myeloid cells compared to normal tissue (9.30% vs. 3.84%) (**Figure 4D**); the 2,334 myeloid transcriptomes represented 7.48% of all cell types. This cluster was defined as myeloid lineage cells based on the pan-expression of *SIRPA*, *CSF1R*, and *FCGR3A* (**Figure 5C**). *SIRPA* and *CSF1R* were recently confirmed to be pan-myeloid lineage markers in the characterization of the porcine immune system (43). However, we observed that markers for more precise subtypes of myeloid cells were not uniform in expression, suggesting that more specific myeloid subtypes could be elucidated. To identify these specific cell types within the myeloid cluster, we subsetted this population from the full dataset and performed principal component analysis (PCA) to generate a new UMAP, identifying eight unique clusters (**Figures 5A, B**). We then generated lists of the top significant DEGs for manual identification and GSEA. Based on the overall higher expression of several MHC Class II molecules, such as *SLA-DRA*, and the lower relative expression of *CD163*, we identified Clusters 6, 7, and 8, as dendritic cells (Dendritic Cells A, Dendritic Cells B, and Dendritic Cells C, respectively) (**Figure 5D**). The remaining clusters had higher relative expression of *CD14* and *CD163* and were identified broadly as macrophages. However, we found that three out of the five macrophage clusters (Clusters 2, 3, and 4) were best classified as M2 macrophages (M2 Macrophage A, M2 Macrophage B, and M2 Macrophage C, respectively), characterized by a higher ratio of *CD206* (M2) to *CD86* (M1) and in some cases, higher expression of macrophage scavenger receptors such as *MSR1* (**Figure 5E**). Cluster 1 was the only macrophage cluster with higher levels of *CD86* and *SLA-DRA* and thus was classified as M1 macrophages (**Figures 5D, E**). Furthermore, M1 macrophages were enriched for GO terms “Response to Cytokine” and “Immune Response,” primarily through the higher expression of *CXCL8, IL1B, CCL5*, and *CXCL16* (**Supplementary Table 4**). Surprisingly, Cluster 5 was defined by high expression of *COL1A1, COL1A2*, and *DCN* while still expressing pan-myeloid markers (**Figure 5F**). While this cluster was the smallest within the myeloid cell population, it was nearly exclusively represented by tumor-derived macrophages (91.53%). Fibrotic macrophages have been characterized in patients with pulmonary fibrosis and correlated with increased mortality and fibrosis (44). Additionally, a recent study using a mouse model of fibrotic scar formation in the heart has shown that monocyte-derived macrophages directly contributed to COL1A1 deposition (45). This cluster might represent a rare population of macrophages with a profound impact on a tumor microenvironment centered around extracellular matrix (ECM) reconstruction.

M2 macrophages represent a critical population in the tumor microenvironment and primarily promote immunosuppressive and pro-fibrotic pathways often found in advanced cancers (46). M2 Macrophage A was enriched for *CCL2, MSR1, CD209, CD206*, and *SCARB2* (**Supplementary Table 4**). GSEA revealed that this cluster, representing 21% of the total myeloid population, was enriched for GO terms “Innate Immune Response” and “Endocytosis,” suggesting a phagocytic M2 macrophage population. M2 Macrophage B was enriched for various chemokines, including *CCL2, CXCL12, CCL19*, and *CXCL14* (**Supplementary Table 4**). In addition to these cytokines, GSEA revealed that M2 Macrophage B was negatively associated with “Immune response” and “Cell Activation,” further suggesting a strong immunosuppressive phenotype (47–51). M2 Macrophage C had a higher relative expression of complement system components, such as *C1QA/B*, and was also negatively associated with “Cellular Response to Biotic Stimulus.” Evidence suggests that complement proteins on macrophages can drive immunosuppression and local tissue remodeling and indicate a poor prognosis (52, 53). Dendritic Cells A is the second largest myeloid cluster and notably was overrepresented by cells derived from tumors (89.69% vs. 83.60% expected, χ^2 = ^18.3, p <0.00005). Cells in this cluster expressed immune checkpoint modulators *IDO1* and *CD274* (*PD-L1)*, thus suppressing T cell activation and providing critical immunosuppressive action in the microenvironment (**Figure 5G**) (54, 55). Furthermore, we observed that *IDO1/PD-L1* expression in the cluster was nearly exclusive to tumor-derived cells (**Figures 5H, I**). Taken together, we provide evidence for M2 macrophage polarization, *IDO1^+^*/*PD*-*L1^+^* dendritic cells, and a novel population of macrophages that combine to provide a strong immunosuppressive tumor microenvironment, further supporting the advanced stage of these neurofibromas.

### Cancer-associated fibroblasts in neurofibromas are pro-inflammatory and express lower levels of type-I collagens

**Figure 6:**
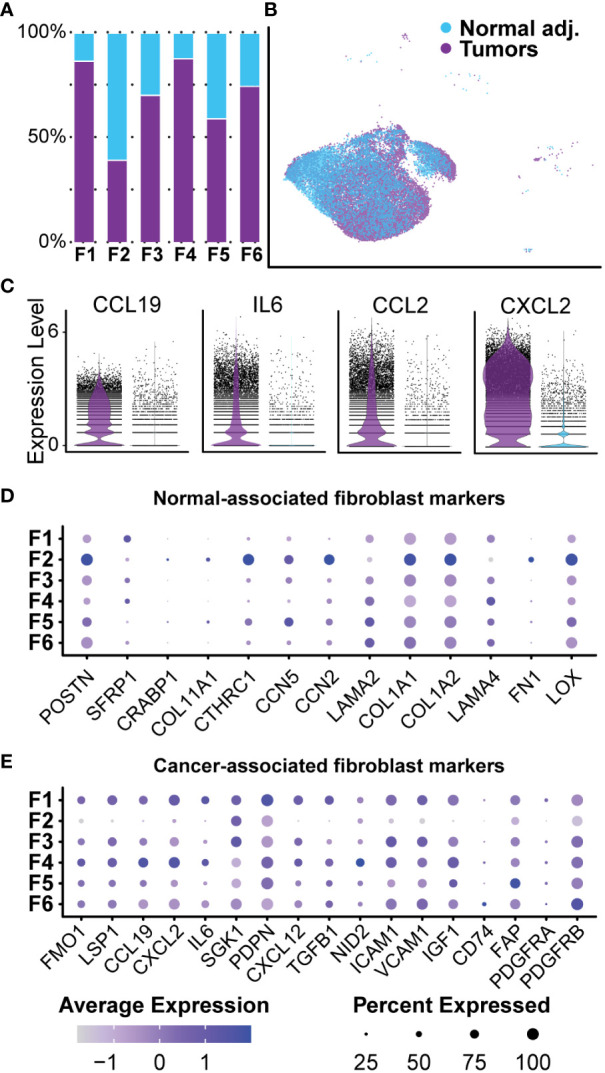
Normal-associated and Cancer-associated Fibroblasts: **(A)** Most clusters were represented by a majority of cells derived from tumors. Cluster 2 was the only cluster that was primarily composed of fibroblasts derived from normal adjacent tissue. **(B)** Fibroblast clusters were separated by sample. **(C)** Each cell is displayed and plotted in arbitrary units. Tumor-derived fibroblast displayed higher expression of inflammatory cytokines *IL-6, CXCL2, CCL19*, and *CCL2*. A violin silhouette of normal tissue is shown but not visible. **(D)** A selection of genes commonly found in normal fibroblasts show enrichment specific to Fibroblast 2. **(E)** A selection of markers typically found in cancer-associated fibroblasts depicts a role for inflammation in Fibroblast 1 and Fibroblast 4. For all dot plots, dot size represents the percentage of cells with expression of the marker and color intensity depicts the average expression level.

Collagen accounts for up to 70% of lipid-free dry-weight in human neurofibromas (56, 57). A recent study of the matrisome of a human cutaneous neurofibroma found that neurofibroma fibroblasts preferentially deposited pro-tumorigenic collagens rather than classical pro-fibrogenic collagens (58). Our current study found that fibroblast-like cells represented the majority (over 70%) of cells in all samples and were positive for canonical fibroblast markers *COL1A1*, *MMP2*, and *DCN* (**Figures 4B, C**). We identified 24,532 transcriptomes derived from fibroblasts and fibroblast-like cells in tumors and normal adjacent tissue, giving us detailed insight into this population. All six clusters were found in all three samples at varying levels of representation. Five clusters (Fibroblasts 1, 3, 4, 5, and 6) were primarily found in the tumors, while Fibroblasts 2 were found mainly in the normal adjacent tissue (**Figure 6A**). However, before analyzing each cluster individually, we separated this population by sample to examine differences between normal adjacent-derived fibroblasts and tumor-derived fibroblasts (**Figure 6B**). While all fibroblasts expressed *COL1A1* at variable levels, fibroblasts in the adjacent normal tissue were significantly enriched for canonical fibroblast markers *CTHRC1*, *CCN2*, *COL1A1*, and *COL1A2* (**Supplementary Table 3**) (59–61). In contrast, fibroblasts derived from the neurofibromas were significantly enriched for *CXCL2*, *IL6*, *CCL19*, and *CCL2* (**Figure 6C**, **Supplementary Table 3**). Top upregulated markers in fibroblasts from the neurofibroma were mostly pro-inflammatory and corresponded to markers of CAFs, while those of normal tissue were associated with resident dermal fibroblasts (**Supplementary Table 3**) (59, 62–64). GSEA of fibroblast-related DEGs revealed that tumor-derived fibroblasts were significantly enriched in processes “Cytoplasmic Translation,” “Peptide Biosynthetic Process,” and “Defense Response” (**Supplementary Table 5**). Neurofibroma-associated fibroblasts were negatively associated with “Collagen Fibril Organization,” “External Encapsulating Structure Organization,” and “Collagen Metabolic Process” (**Supplementary Table 5**). The matrisome from the porcine neurofibromas recapitulates that of human neurofibromas described by Brosseau and colleagues (58).

### Neurofibroma-associated fibroblast clusters express unique collagens and glycoproteins favoring ECM reconstruction and nerve regeneration

**Figure 7:**
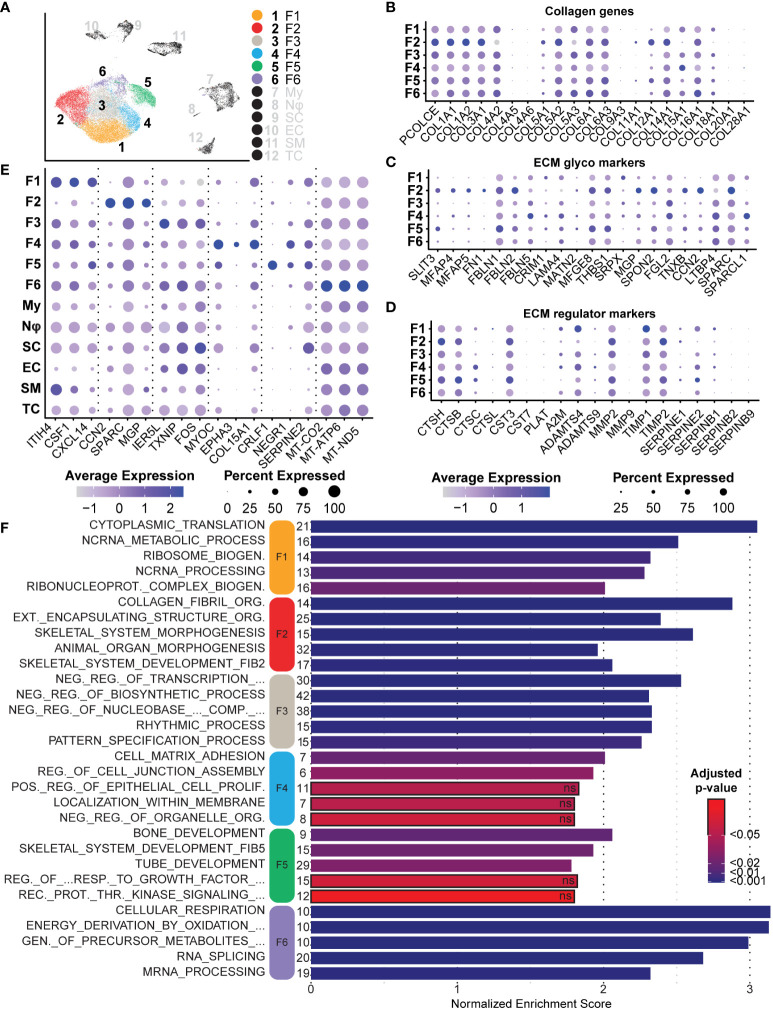
Analysis of Fibroblasts and Roles in the Tumor Microenvironment: **(A)** Fibroblast clusters were subset and separated, depicting six unique clusters. **(B)** Relative expression of collagens shows enrichment primarily in Fibroblast 2. Notable exceptions include *COL4A2* and *COL15A1*. **(C)**. Relative expression of ECM glycoprotein genes shows enrichment in Fibroblast 2, except for *FBLN5* and *LAMA4*, both associated with processes of nerve regeneration. **(D)** Relative expression of ECM regulator genes depicts the enrichment of several cathepsin genes in Fibroblast 5, suggesting a role of Fibroblast 2 and 5 in ECM turnover. **(E)** A selection of notable DEGs from each cluster representative of specialized roles or features. **(F)** DEGs for each cluster were used for GSEA analysis. The top 5 enriched processes for each cluster reveal unique roles. Bars outlined in black were not significant. The number of genes in the Leading Edge driving the enrichment for processes are shown to the left of each bar. The Normalized Enrichment Score is shown on the x-axis. For all dot plots, dot size represents the percentage of cells with expression of the marker and color intensity depicts the average expression level. .

After bulk comparisons of fibroblasts in the tumors and normal adjacent tissue, we explored each fibroblast cluster individually to elucidate fibroblast diversity. While this large group of cells can be placed under the umbrella of fibroblasts, initial data integration and analysis split the group into six distinct clusters (**Figure 7A**). As previously noted, we found that Fibroblast 2 was the only cluster represented by a majority of normal-derived fibroblasts, while Fibroblasts 1, 3, 4, 5, and 6 were represented by a majority of tumor-derived fibroblasts. Functionally, normal-associated fibroblast markers were enriched in Fibroblast 2, while cancer-associated markers were enriched in the remaining five clusters (**Figures 6D, E**). Because collagens are critical components of the neurofibroma matrisome, we examined the expression levels of all detected collagens in each fibroblast cluster. Fibroblasts 2 had significantly higher expression of most collagens analyzed in this study, highlighting the importance of that cluster in ECM construction (**Figure 7B**). Fibroblasts 2 was enriched for *PCOLCE*, a pro-collagen that enhances reconstruction of the ECM, as well as some of the most abundant pro-fibrotic collagens found in the body, *COL1A1* and *COL1A2* (**Figure 7B**) (65). However, additional noteworthy collagens were higher in the other clusters, perhaps highlighting a specialized role for each group. *COL5A3*, which promotes Schwann cell adhesion and neurite growth (66, 67), was significantly higher in Fibroblasts 6. *COL4A2* was also enriched in Fibroblasts 6; Type IV collagens are localized to basement membranes and surround Schwann cells to promote axonal growth (66). Finally, *COL15A1*, a critical component in nerve regeneration (68, 69), was enriched in Fibroblasts 4 (**Figure 7B**). With this data, we hypothesize that Fibroblasts 2 is the primary driver of ECM deposition, while Fibroblasts 4 and 6 may play a more specialized role in supporting processes of nerve regeneration. Notably, 87.8% of Fibroblasts 4 and 74.7% of Fibroblasts 6 were found in the neurofibromas (**Figure 6B**).

We next examined the expression of several major ECM glycoproteins and ECM regulatory proteins (58). With a few exceptions, we found that Fibroblasts 2 was enriched for most major ECM glycoproteins, including *MGP, CCN2*, and *SPARC* (**Figure 7C**) (70, 71). Notable exceptions include *FBLN5* and *LAMA4*, most highly expressed in Fibroblasts 4 (**Figure 7C**). *FBLN5* is a critical factor in promoting Schwann cell proliferation and myelination, while *LAMA4* works in combination with *COL15A1* to promote nerve regeneration (72). This further suggests that Fibroblasts 4 may represent a specialized group of fibroblasts within the neurofibroma microenvironment that promotes nerve regeneration and maintenance processes. Fibroblasts 2 was enriched for most of the queried ECM regulatory protein genes, while Fibroblasts 5 was enriched for several cathepsins (**Figure 7D**). Cathepsins are proteases involved in the maintenance of ECM by promoting the turnover of extracellular proteins (73). Taken together, we hypothesize that Fibroblasts 2 and 5 primarily promote ECM reconstruction and maintenance, while Fibroblasts 4 and 6 are more specialized for nerve regeneration and maintenance.

### Tumors contain distinct groups of fibroblasts with unique roles in the microenvironment

GSEA was performed on each fibroblast cluster to examine critical pathways potentially driving tumor progression or tumor microenvironments. To elucidate roles unique to each fibroblast cluster in the context of the microenvironment, we compared each to all other cells in the dataset, including non-fibroblast cells. All significant upregulated and downregulated DEGs with a minimum log-fold change of 0.25 were then analyzed using the *fgsea* package against GO-Biological Processes (GOBP) gene sets. This analysis revealed distinct enriched pathways for each cluster of fibroblasts, further highlighting each cluster’s unique role in the tumor microenvironment beyond matrisome function.

Fibroblasts 1, a cluster composed primarily of tumor fibroblasts (86.65%), was highly enriched for ribosomal L and S mRNAs (**Supplementary Table 3**) and other genes associated with translation and ribosome biogenesis (**Figure 7F**) (74). The significance of increased protein translation is difficult to pinpoint, but heightened translation is a critical component of tumorigenic and differentiating cells, suggesting this is a highly active population (75, 76). Dysregulation of mRNA translation is a major feature of neoplasia and stem cell maintenance (77–79). Plasticity is often a consequence of cells adapting to the tumor microenvironment, especially hypoxia, nutrient limitation, or cancer therapeutics, and may reflect an important role of change in translation in phenotypic switching of cancer cells (75, 80). Additionally, ribosomal proteins can have functions outside of translation, including induction of cyclins and NFKβ (81–83). Notable overexpressed genes for Fibroblasts 1 include *IL-6, CSF-1, CXCL12, ITIH4*, and *CXCL14*, all factors that promote inflammatory responses and commonly found in CAFs (**Figure 7E**, **Supplementary Table 3**) (84–89). *CSF1* is one of the ligands for *CSF1R* on macrophages, and upon binding, *CSF1* promotes M2 polarization of macrophages. This suggests that Fibroblasts 1 may be partly responsible for macrophage class switching and immune evasion.

Fibroblasts 2, the only cluster predominantly composed of fibroblasts from adjacent normal tissue, was highly enriched for dozens of collagens and ECM regulatory proteins, as noted in the previous section. Consequently, the top three significant non-redundant GOBP processes enriched for this cluster were “Collagen Fibril Organization,” “External Encapsulating Structure Organization,” and “Animal Organ Morphogenesis” (**Figure 7F**). Many of the top overexpressed genes include the previously discussed collagens, but also cell-cell communication molecules *CCN2, SPARC*, and *MGP* (**Figure 7E**) (71, 90, 91). Interestingly, this cluster also overexpressed *THY-1* and *SPON2.* While *THY-1* and *SPON2* can be expressed in normal fibroblasts and smooth muscle cells, they are also associated with synaptogenesis, neuron outgrowth, and tumorigenesis. They are often found in other nervous system cell types (92–96).

Spatially, Fibroblasts 3 was surrounded by the other five fibroblast clusters and was generally non-descriptive in enriched processes and genes. The top 3 enriched pathways relate to negative regulation of transcription, while other significant non-redundant processes include the “Rhythmic Process” and “Pattern Specification Process” (**Figure 7F**). A decreased rate of transcriptional activity is associated with senescence, differentiation, apoptosis, angiogenesis, and neoplastic transformation (97, 98). Although chromatin exposure and high transcription rates are found in embryonic stem cells, upon initiation of differentiation, cells acquire markers for transcriptional repression. A recent meta-analysis of human lung cancer samples in The Cancer Genome Atlas found that genes represented in the “Rhythmic Process” were associated with immune activation, circadian rhythm, and carcinogenic pathways (99). With this information in combination with enriched processes of pattern specification, regionalization, and various organ development processes, we hypothesize that this population represents a dedifferentiated group of fibroblast-like cells that are more involved in the tumor microenvironment than the GOBP terms suggest.

Fibroblasts 4 represented the highest proportion of tumor-derived cells within a single cluster at 87.75% (**Figure 6B**). This cluster was unique because many top upregulated genes are associated with neural development or nerve regeneration. These genes included *NEGR1, MYOC, EPHA3, COL15A1, PTN, FBLN5, PDGFRβ*, and *THY-1* (**Figure 7E**, **Supplementary Table 3**) (72, 94, 100–105). In addition to these genes, this cluster was enriched for both “Cell-Matrix Adhesion” and “Regulation of Cell Junction Assembly” (**Figure 7F**). These two processes are essential in the development of signaling networks and neural regeneration. Specifically, *EPHA3* and *NEGR1* are primarily expressed in the adult brain and promote neuronal synaptogenesis and neurite outgrowth (106, 107). Loss of *NEGR1* in development is associated with decreased numbers of synapses and dendritic length, resulting in anxiety and depression-like behaviors in mice (100). Furthermore, *FBLN5* and *PDGFRβ* are found to be upregulated immediately following sciatic nerve crushing in rats. It has been recently reported that *FBLN5* promotes the proliferation of Schwann cells *in vitro* (72, 105). We hypothesize that this cluster is profoundly important in promoting a hospitable environment for neurofibroma development.

Like Fibroblasts 4, Fibroblasts 5 also expressed genes associated with the peripheral and central nervous systems. These markers include *CRLF1, SERPINE2, NEGR1, GRIA2, NRXN1, SOX9*, and *LGALS3* (**Figure 7E**, **Supplementary Table 3**) (100, 108–113). While both these clusters express neural markers, the processes they influence are distinct. Specifically, Fibroblasts 4 expresses markers consistent with promoting cell-cell interactions and processes necessary for synapse formation, but Fibroblasts 5 is enriched for several processes of early neuronal development (**Figure 7F**, **Supplementary Table 5**). Perhaps the most notable of these processes is the enrichment of “Tube Development.” While many of the included genes in this set pertain to processes associated with angiogenesis and uretic bud formation, it should be noted that many of these markers have dual roles reliant on context. Specifically, the expression of mRNAs such as *SFRP2* and *GRIA2* in this cluster may favor smaller sub-processes related to the neural tube or neural development processes, such as “Gliogenesis” (114–116).

Finally, Fibroblasts 6, the smallest group among the six clusters and similar to Fibroblasts 3, has few notable features or upregulated processes. The top processes enriched were primarily driven by overexpression of mitochondrial-derived genes such as *MT-ND5*, despite that we excluded cells with over 10% representation of mitochondrial genes (**Figure 7E**). Mitochondrially-derived upregulated factors in Fibroblasts 6 favored the enrichment of several redundant processes related to cellular respiration and metabolism (**Figure 7F**). Excluding mitochondrial mRNAs, this cluster was also significantly enriched for processes affecting RNA splicing and mRNA processing (**Figure 7F**). This cluster is notably post-translationally and metabolically active, features common to cells undergoing differentiation (117, 118). Taken together, we provide evidence that each group of fibroblasts is specialized for a unique role in the tumor microenvironment. Despite high expression of neuronal-associated markers in some fibroblast clusters, all were negative for neural crest lineage marker *SOX10* (**Figure 4C**) (119–121). The absence of this lineage marker suggests these classes did not arise from neural crest stem cells but may be resident or recruited fibroblasts reprogrammed for the nerve regeneration microenvironment by tumorigenic Schwann cells. Further, this supports the possibility that cell signaling pathways associated with nerve regeneration are drivers of neurofibroma formation (122).

### Schwann cells express precursor markers and reprogram the tumor microenvironment for neural regeneration

**Figure 8:**
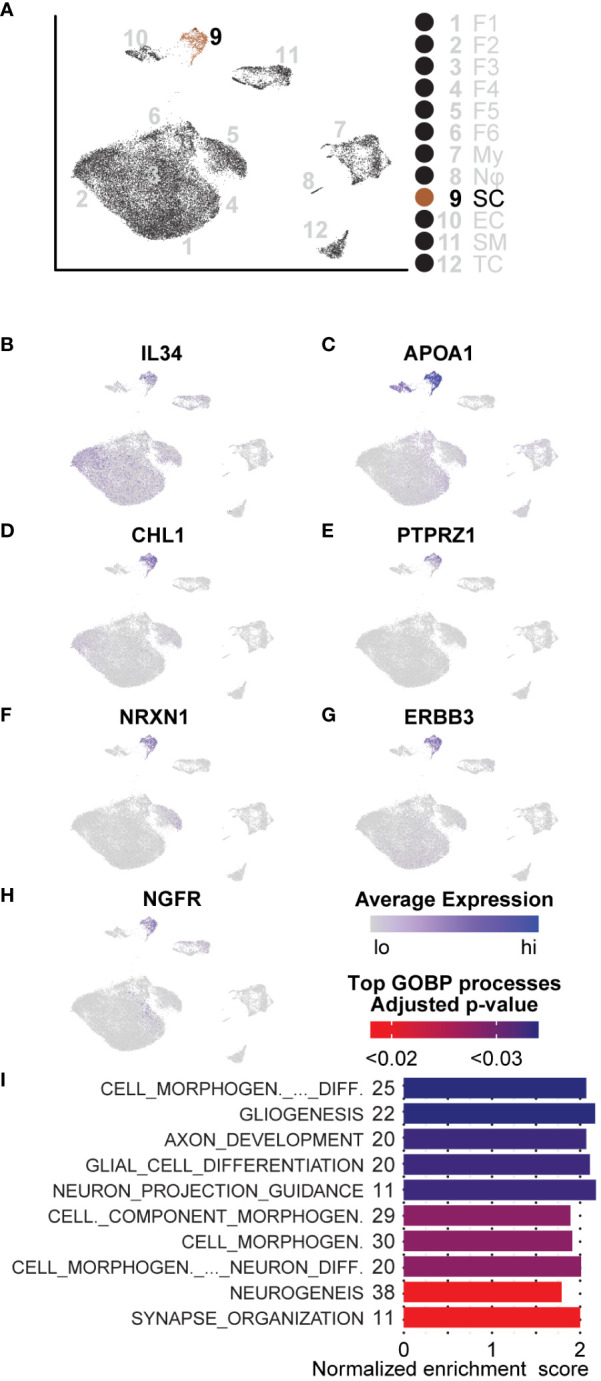
GSEA and DEGs of Schwann cells are indicative of an immature state: **(A)** UMAP depicting Schwann cell cluster to be analyzed. DEGs for GSEA of Schwann cells were derived by comparing SCs to all other cells in the dataset. **(B)** Relative expression of IL-34, a potent M2 macrophage polarization molecule. **(C–H)** Relative expression of *APOA1, CHL1, PTPRZ1, NRXN1, ERBB3*, and *NGFR* indicative of an immature Schwann cell or processes related to nerve regeneration in Schwann cells. **(I)** The top GOBP processes that were found to be enriched in Schwann cells. The number of genes represented in the Leading Edge are depicted to the left of the Normalized Enrichment Score bars. All processes are associated with morphogenesis, nerve regeneration, or development.

We characterized Schwann cells using the same analysis methods described for the aforementioned cell types (**Figure 8A**). Because of the distinct lack of Schwann cells in the adjacent tissue, comparisons between tumorigenic and non-tumorigenic Schwann cells were not possible from this dataset. Instead, we compared Schwann cells universally and used DEGs to identify enriched pathways. The top three processes enriched in Schwann cells were all associated with processes of development and repair: “Cell Morphogenesis Involved in Differentiation,” “Gliogenesis,” and “Axon Development.” Beyond these top three pathways, the top ten enriched pathways were all related to cell morphogenesis or neural development processes (**Figure 8I**). Within these top ten pathways, there were 59 unique genes upregulated, which accounted for 20.4% of all significantly enriched Schwann cell genes. To confirm that identified pathways were not biased by comparing Schwann cells to non-Schwann cells, which would inherently highlight neuronal pathways, we queried genes representing enriched processes in the Sciatic Nerve Atlas (123). Within the top three enriched gene sets, we found that 52% of the genes displayed maximum expression in immature Schwann cells, which we defined as peak expression occurring in Schwann cells harvested no later than E13.5 or E17.5. Enriched genes representative of immature Schwann cells includes *APOA1, CHL1, NRXN1, NGFR, ERBB3*, and *PTPRZ1* (**Figures 8C–H**). Interestingly, each one of these factors is also associated with various stages of Schwann cell activity during nerve injury (69, 111, 124–129). Other notable enriched mRNAs indicative of a nerve regeneration/repair phenotype include *SPP1* and *CLU*, two secreted factors that promote motor and sensory neuron regeneration, respectively (130).

We also found that roughly 60% of Schwann cells in the microenvironment had significantly higher expression of *IL-34*, recently discovered to be a second ligand for the macrophage receptor, *CSF1R*; *IL-34* binding to *CSF1R* potently promotes M2 macrophage polarization (**Figure 8B**) (131, 132). Given that we observed an overall enrichment of M2 macrophages in the tumor microenvironment, this data suggests that neurofibroma-associated Schwann cells may directly promote this polarization and, therefore, directly contribute to reprogramming the microenvironment to be immunosuppressive. Schwann cells are unique in the ability to de-differentiate from a fully differentiated mature state to an immature state following nerve injury (133–135). During this process, an inflammatory response is involved; Schwann cells proliferate, immune cells and other fibroblasts are recruited, and the ECM composition is modified (136). Once this process is complete, Schwann cells stop proliferating and become mature myelinating cells again (133). Considering processes occurring in other cell types, including ECM modifying fibroblasts and immunosuppressive functions of myeloid cells, we conclude that this neurofibroma is under a state of dysregulated nerve repair. The presence of immature Schwann cells, immunosuppressive myeloid cells, multiple types of fibroblasts, and modified ECM deposition supports this notion.

## Conclusions

**Figure 9:**
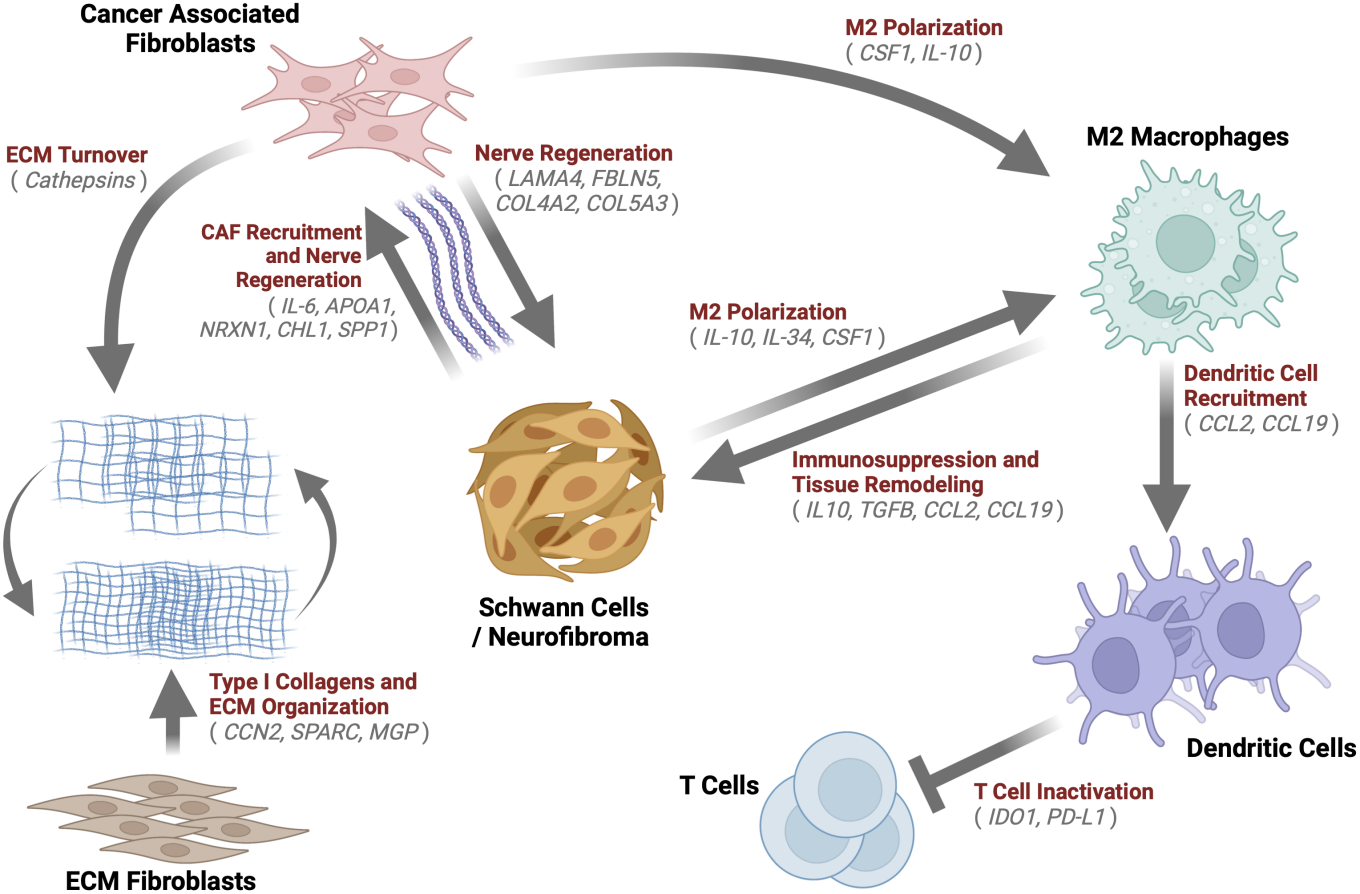
Neurofibroma Microenvironment: A graphical description of the extensive crosstalk uncovered within the Neurofibroma microenvironment. This study identified notable enriched genes (grey) for multiple cell types. We hypothesize the inter-cell type interactions within neurofibromas depicted by arrows, which have been demonstrated in other studies. These interactions serve to recruit CAFs (85, 111, 124, 125, 130, 137), induce immunosuppression (47, 64, 138, 139), promote ECM Organization and turnover (61, 71, 73, 91), M2 macrophage polarization (131, 139), T Cell inactivation (140, 141), and tissue remodeling (47, 64, 138, 139). Schwann cells were found to be similar to immature Schwann cells. Taken together, this tumor microenvironment, reprogrammed by immature SCs, is akin to nerve regeneration environments.

This paper aimed to determine the composition and transcriptional biology of cells within the tumor microenvironment of mature spontaneous neurofibromas in a porcine model of NF1. The main findings of the study are that porcine mature neurofibromas closely resemble human neurofibromas histologically and harbor all known cellular components of their human counterparts. Gene expression data reveals a heterogeneous tumor microenvironment that is enriched for processes of immunosuppression, M2 macrophage polarization, ECM remodeling, and nerve regeneration. We also observed the presence of unique cells, such as fibrotic macrophages, that have not been reported in NF1. We also, for the first time, report a large population of dendritic cells concurrently overexpressing two check-point proteins, IDO1 and PD-L1. In **Figure 9**, we present a model of the neurofibroma microenvironment suggested by our findings.

The neurofibromas in our porcine model selected for the study were immunoreactive for the two predominant neurofibroma diagnostic proteins, *S100* and *CD34*; Masson’s Trichrome staining showed heavy collagen deposition with a large population of spindle-shaped cells and immune infiltrate (**Figure 2**). Initial analysis revealed that these porcine tumors contained all types of cells commonly found in neurofibromas. Within the neurofibromas, 4.37% of cells were Schwann cells, while 79.8% were fibroblasts and fibroblast-like cells (**Figure 4D**). A recent study comparing the human pNF microenvironment to that of a genetically engineered mouse model pNF found 0.7% of the microenvironment to be Schwann cells in humans while 14% in mice (142). Human cutaneous neurofibromas are 3.16% Schwann cells, and appear to contain a much higher percentage of immune and stromal cells than their mouse counterparts (58). At least in this context, these porcine neurofibromas appear to be more similar to those in humans. While we noted a few interspersed cells histologically resembling mast cells within the tumor, we could not identify mast cells by gene expression signature. We hypothesize that mast cells comprise a small portion of the larger group of myeloid cells identified. Of the two recent publications that profiled porcine peripheral immune cells, one could not identify mast cells, while the other only identified a very small population in the intestinal tract (43, 143). More robust transcriptomic markers for the identification of porcine mast cells may be needed. Alternatively, in our porcine samples, mast cells may be absent or in low abundance and thus may reflect the type of neurofibromas or their maturity.

Over the years, proposed mechanisms of neurofibroma initiation and progression have highlighted the prominent role of mast cells (144). *NF1^-/-^* Schwann cells have been shown to secrete pathologic concentrations of stem cell factor (SCF) that signal the recruitment of *NF1^+/-^* mast cells via a cKIT-dependent pathway (144, 145); *NF1^+/-^* mast cells were observed to be hypersensitive to SCF (146). As mast cells have been implicated in the growth, vascularization, and spread of neoplastic conditions (144), investigators studying NF1 have increasingly sought to elucidate the role of mast cells within neurofibromas. While studies have implicated the importance of mast cells in NF1 tumor progression (147), others have illustrated that their involvement, at least in a spatial context, may differ depending on tumor type. Histological analysis of diffuse neurofibromas and encapsulated neurofibromas found that mast cells were abundant in diffuse neurofibromas but were absent or excluded to the periphery in encapsulated tumors (148). It has also been observed that more advanced neurofibromas have markedly decreased mast cell populations and that mast cell presence has no prognostic value (148–152). Considering that our porcine tumors were not visibly encapsulated, and gene expression patterns indicate a more advanced phenotype, we hypothesize that mast cells were significantly diminished (or absent) by the time this tumor was studied. In addition, as our samples represented the interior area of the tumors, it is possible that mast cells on the periphery were missed. The role of mast cell signaling for the formation and early maintenance of neurofibromas warrants scrutiny in future studies.

The importance of an injury-like environment in neurofibroma initiation and progression has been previously highlighted (122, 142). The peripheral nervous system has a remarkable ability to repair damaged nerves, a process that hinges upon the dedifferentiation of Schwann cells (133, 138). In peripheral nerve injury, the loss of contact of Schwann cells with axons is thought to be an essential step in driving Schwann cell dedifferentiation. Disruption of *NF1* in Schwann cells has been shown to be sufficient in disrupting Schwann cell/axonal interactions (7, 122, 153). Nerve injury signals seem to be required for neurofibroma formation; *NF1^-/-^* myelinating Schwann cells did not form neurofibromas unless placed at a nerve injury site (122). While neurofibromas can develop in both *NF1^+/+^* and *NF1^+/-^* microenvironments (122), a recent study found that a heterotypic population of differentiating *NF1^-/-^* Schwann cells and *NF1^+/-^* pNF-derived fibroblasts provided the most potent cell mixture for generating pNFs *in vitro* and *in vivo* (154). RNA-seq analysis revealed that pNFs generated from these cells were composed of Schwann cells at multiple stages of the neural crest-Schwann cell differentiation axis, a phenomenon common to nerve regeneration. Similarly, our porcine dpNFs were also found to express factors associated with various stages of Schwann cell differentiation during nerve injury. Examination of DEGs from Schwann cells in this study revealed enrichment of genes and processes associated with nervous system development, nerve regeneration, and M2 macrophage polarization. More specifically, the neurofibroma-associated Schwann cells displayed markers of dedifferentiated Schwann cells and promoted processes associated with synaptogenesis, axon elongation, and neurogenesis (**Figure 8**).

The dedifferentiation of Schwann cells upon peripheral nerve injury has been previously observed *in vivo* (133). These Schwann cells then actively recruit immune cells through the expression of pro-inflammatory cytokines and begin demyelination, a process known as Wallerian degeneration. The success of peripheral nerve repair relies on signals from Schwann cells produced when they come into contact with the severed axon stump. If the appropriate signals are not produced, Schwann cells may not recover from a regenerative state and thus would continue to proliferate and promote a favorable environment for tumorigenesis. We found evidence that sub-populations of neurofibroma-associated fibroblasts further support this process. In this context, we provide evidence that NF1^-/-^ Schwann cells may be trapped in a process similar to that found in peripheral nerve regeneration.

Fibroblasts were the largest and most transcriptionally diverse group within our tumors. Over 70% of all samples analyzed here were composed of fibroblast-like cells that clustered into six distinct groups upon initial dimensional reduction and UMAP analysis. DEGs were distinct between those associated with tumors compared to those in adjacent normal tissue. We found that neurofibroma-associated fibroblasts had significantly higher expression of inflammatory cytokines and CAF genes that stimulate the recruitment of additional cells to the tumor and promote ECM remodeling. Conversely, the normal tissue-associated fibroblasts had higher expression of canonical markers such as *CTHRC1, CCN2, COL1A1*, and *COL1A2* (**Figure 6D**). Decreased expression of Type I collagens has been observed in human neurofibromas, and we show that to hold true in the porcine NF1 model (58). Our examination of fibroblasts subclasses reveals that Fibroblasts 2, the most heavily represented class in normal adjacent tissue and associated with pro-fibrotic processes and higher expression of most collagens, was significantly reduced in the tumor microenvironment (**Figure 7B**). This is consistent with the observation that collagens such as *COL1A1* and *COL1A2*, often associated with pro-fibrogenic skin fibroblasts, are downregulated in human neurofibromas (58, 155). ECM composition containing Type I collagens might be less conducive to tumor growth. In contrast, collagens such as *COL4A2* (66)*, COL5A3* (66, 67), and *COL15A1* (68, 69), which play roles in axonal growth, Schwann cell adhesion and neurite growth, or are critical components in nerve regeneration, were enriched in fibroblast groups primarily found in the two dpNFs **(** **Figure 7B** **)**.

In addition to their potential role in altering the ECM structure within the microenvironment, certain groups of fibroblasts seem to contribute to an environment of immunological activity and nerve regeneration. Fibroblasts 1, the most abundant fibroblast type in the dpNFs, represented CAFs that promote immune cell recruitment by producing *IL-6, CCL19, CXCL2*, and *TGFB*, and inflammation by other additional factors such as *CSF-1, CXCL12*, and *CXCL14* (**Figure 7E**). *IL-6* was heavily expressed by a majority of the fibroblast populations in the neurofibroma; in addition to its role in inflammation, it is thought to be an early indicator of nerve injury (137) (**Figure 6C**). Interestingly, we identified two clusters of fibroblasts (Fibroblasts 4 and 5) that expressed markers of neuronally derived cells, such as *FBLN5, EPHA3, NEGR1, NRXN1*, and *THY1* (**Figure 7E**). Enriched processes in these fibroblasts were related to early development, cell junction assembly, and synaptogenesis, which are critical in peripheral nerve regeneration and neural development (**Figure 7F**). We also identified two groups of fibroblasts (Fibroblasts 3 and 6) undergoing transformation or reprogramming. During development, neural crest stem cells undergo multilineage differentiation to generate both endoneurial fibroblasts and Schwann cells (156). We also identified numerous markers of early Schwann cells within these fibroblast subpopulations. However, these fibroblasts expressed little to no *SOX10* or *NGFR*, markers of cells derived from neural crest stem cells (**Figure 4C**, **8H**) (120, 121). Therefore, we hypothesize that this large population of fibroblasts are actively being recruited and reprogrammed by *NF1^-/-^* Schwann cells in a process similar to that which occurs in nerve regeneration. Fibroblasts 6, defined by high RNA splicing and mRNA processing, may represent a subpopulation in a transitional or differentiating stage (**Figure 7F**).

Immune evasion is considered a hallmark of advanced tumors or cancers and is a major hurdle to therapeutically overcome. Tumors evade attacks from the immune system through several mechanisms, including restriction of antigen recognition, recruitment of immunosuppressive cells, induction of T-cell exhaustion, or blocking of T-cell activation (157). Immune profiling of neurofibromas has been very limited and has mainly been focused on analyzing the role of mast cells in tumor initiation and progression (144). Immune profiling of NF1-associated tumors by Haworth and colleagues highlighted the importance of understanding the immunogenicity of these tumors, especially in the context of tumor heterogeneity (158). In our study, we identified specific populations of myeloid cells potentially contributing to an immune-evasive tumor microenvironment. We detected M2 macrophages, M1 macrophages, dendritic cells, and a rare group of fibrotic macrophages (**Figure 5**).

Macrophages are thought to promote the growth of established pNFs and studies have shown macrophage density increases with tumor progression to MPNST (159). Consistent with these findings, we found M2 macrophages specializing in phagocytosis or immunosuppression through increased expression of cytokines such as *IL-10* in our established porcine dpNFs. In these cells, both *IL-10* and *ARG1*, act to block T-cell proliferation and promote the conversion of inflammatory monocytes (M1) to immunosuppressive monocytes (M2) (139, 160). M2 macrophages can directly inhibit the activation and proliferation of T-cells, promote fibrosis, and are significant contributors to resistance to therapies (161, 162). This polarization to M2 macrophages is also compounded by fibroblast expression of *CSF1* and Schwann cell expression of *IL-34* (131). We found overexpression of *CSF1* and *IL-34* by a subset of fibroblasts (Fibroblasts 1) and 60% of Schwann cells, respectively, in the porcine dpNFs.

A cluster of dendritic cells, exclusive to the dpNFs, were enriched for immune checkpoint modulators, *IDO1* and *PD-L1*, which also act to impair CD8^+^ T cells while also inhibiting AMPK signaling, a tumor suppression pathway (54, 163) (**Figure 5H, I**). Programmed cell death-ligand 1 (PD-L1) is known to induce inhibitory signals through interaction with programmed cell death protein 1 expressed on the cell surface of T cells, which results in suppression of tumor-specific T cell response. This mechanism plays a vital role in the process of tumor immune tolerance and immune escape (140). Resistance to cell-mediated immunity through *PD-L1* is a phenomenon that has been studied thoroughly in the cancer biology field (164) and has more recently been examined in human NF1 samples where researchers found histological positivity in 11 of 12 pNFs, suggesting a role in the advancement of the tumors (165).

While upregulation of PD-L1 in pNFs and MPNSTs has been recently noted (165), to our knowledge, the current study is the first to observe an overexpression of (IDO1) in neurofibromas. IDO1 is an intracellular, immunosuppressive rate-limiting enzyme in the metabolism of tryptophan to kynurenine (141, 166). Increased expression of IDO1 is observed in many tumors, including colorectal, hepatocellular, ovarian, and melanomas (167–170). The high expression and activity of IDO1 lead to “tryptophan starvation” in the cell microenvironment. Depletion of tryptophan inhibits T-cell proliferation (171). The main metabolite of tryptophan degradation, kynurenine, also has a direct toxic effect on T-cells and induces T-cell apoptosis. Kynurenine, a natural ligand for aryl hydrocarbon receptors, can regulate the differentiation direction of Th17/Treg cells, thereby promoting the balanced differentiation of Th17/Treg to Treg cells to stimulate anergy of effector T-cells, while Treg activity is enhanced. In the local tumor microenvironment, CTLA-4 expression in Tregs upregulates IDO1 in DCs, which reciprocally promotes Treg activation. Besides suppressing anti-tumor immune responses, tumoral IDO1 is involved in tumor vascularization and lymphangiogenesis (141). Tumoral IDO1 is collectively thought to be a modulator that bridges inflammation, vascularization, and immune escape to promote primary and metastatic tumor outgrowth. Tumors with high expression of IDO1 tend to increase metastatic invasion and have poor clinical outcomes in cancer patients. IDO1 is considered to be a new target for tumor therapy, and inhibition of IDO1 activity by using IDO1 inhibitors can increase patient survival (172).

In conclusion, we have provided evidence that the neurofibroma microenvironment has many parallels to the peripheral nerve injury environment, and perhaps nerve injury acts as a catalyst for neurofibroma development (**Figure 9**). This work represents the first histological and transcriptomic verification of porcine neurofibromas. We reveal an advanced neurofibroma microenvironment that promotes and favors nerve regeneration and propose that this dysregulated process drives the establishment and progression of neurofibromas. The natural progression of the neurofibromas to a stage of advanced immune evasion, evidenced by co-expression of *IDO1* and *PD-L1*, indicates that porcine NF1 models may be an ideal platform for the study of the biology of neurofibroma advancement and the development of therapies that effectively combine checkpoint inhibitors.

## Data availability statement

The datasets presented in this study can be found in online repositories. The names of the repository/repositories and accession number(s) can be found below: https://www.ncbi.nlm.nih.gov/, SUB13587360.

## Ethics statement

The animal study was approved by Institutional Animal Care and Use Committee University of Wisconsin-Madison. The study was conducted in accordance with the local legislation and institutional requirements.

## Author contributions

DM: Supervision, Data curation, Formal Analysis, Investigation, Methodology, Project administration, Software, Visualization, Writing – original draft, Writing – review & editing. JM: Conceptualization, Investigation, Methodology, Project administration, Writing – review & editing. LL: Data curation, Formal Analysis, Visualization, Writing – review & editing. DS: Methodology, Writing – review & editing, Investigation, Conceptualization. DP: Data curation, Writing – review & editing, Supervision. TD: Conceptualization, Investigation, Writing – review & editing, Methodology. DB: Data curation, Formal Analysis, Validation, Writing – review & editing. PS: Formal Analysis, Software, Validation, Writing – review & editing. MG: Data curation, Validation, Writing – review & editing, Formal Analysis, Methodology. LL: Data curation, Validation, Writing – review & editing, Formal Analysis, Methodology. KG: Investigation, Methodology, Writing – review & editing. JR: Methodology, Writing – review & editing, Supervision, Resources. SB-D: Investigation, Methodology, Writing – review & editing, Supervision. CK: Conceptualization, Funding acquisition, Project administration, Resources, Supervision, Writing – review & editing. SR-K: Supervision, Validation, Visualization, Writing – review & editing. DS: Conceptualization, Data curation, Formal Analysis, Funding acquisition, Investigation, Methodology, Project administration, Resources, Visualization, Writing – original draft, Writing – review & editing, Supervision. CR: Conceptualization, Data curation, Formal Analysis, Investigation, Methodology, Project administration, Resources, Supervision, Validation, Visualization, Writing – original draft, Writing – review & editing, Funding acquisition.
