# Supplementary material for: Single-cell RNA sequencing of neurofibromas reveals a tumor microenvironment favorable for neural regeneration and immune suppression in a neurofibromatosis type 1 porcine model
Source: Front Oncol. 2023 Sep 25;13:1253659. doi: 10.3389/fonc.2023.1253659 (PMC10561395; doi:10.3389/fonc.2023.1253659)
Supplement: Supplementary file 1 [file DataSheet_1.pdf]

Gene Symbol	Gene Name
ACTA2 (SMA)	actin alpha 2, smooth muscle
AIF1	Allograft inflammatory factor 1
APOA1	Apolipoprotein A1
ARG1	Arginase 1
C1QA	Complement C1q A chain
C1QB	Complement C1q B chain
CCL19	C-C motif chemokine ligand 19
CCL2	C-C motif chemokine ligand 2
CCL5	C-C motif chemokine ligand 5
CCN2	Cellular communication network factor 2
CD117 (cKIT)	KIT Proto-oncogene
CD14	CD14 molecule
CD16 (FCGR3A)	Fc Gamma Receptor IIIa
CD163	CD163 molecule
CD204 (MSR1)	Macrophage Scavenger Receptor 1
CD206 (MRC1)	Mannose Receptor C-Type 1
CD209	CD209 molecule
CD274 (PD-L1)	Programmed Cell Death 1 Ligand 1
CD34	CD34 Molecule
CD3D	CD3 delta subunit of T-cell receptor complex
CD3E	CD3 epsilon subunit of T-cell receptor complex
CD86	CD86 molecule
CDH5	Cadherin 5
CHL1	Cell adhesion molecule L1 like
CLU	Clusterin
COL15A1	Collagen type XV alpha 1 chain
COL1A1	Collagen type I alpha 1 chain
COL1A2	Collagen type I alpha 2 chain
COL4A2	Collagen type IV alpha 4 chain
COL5A3	Collagen type V alpha 3 chain
CRLF1	Cytokine receptor like factor 1
CSF1	Colony stimulating factor 1
CSF1R	Colony stimulating factor 1 receptor
CTHRC1	Collagen triple helix repeat containing 1
CTLA-4	Cytotoxic T-Lymphocyte associated protein 4
CXCL12	C-X-C motif chemokine ligand 12
CXCL14	C-X-C motif chemokine ligand 14
CXCL16	C-X-C motif chemokine ligand 16
CXCL2	C-X-C motif chemokine ligand 2
CXCL8	C-X-C motif chemokine ligand 8
DCN	Decorin
DES	Desmin
EPHA3	EPH receptor A3
ERBB3	Erb-B2 receptor tyrosine kinase 3
FBLN5	Fibulin 5
GFAP	Glial fibrillary acidic protein

GPR183	G protein-coupled receptor 183
GRIA2	glutamate ionotropic receptor AMPA type subunit 2
IDO1	Indoleamine 2, 3-dioxygenase 1
IL-10	Interleukin 10
IL1B	Interleukin 1 beta
IL-34	Interleukin 34
IL6	Interleukin 6
ITIH4	Inter-alpha-trypsin inhibitor heavy chain 4
LAMA4	Laminin subunit alpha 4
LGALS3	Galectin 3
MGP	Matrix Gla protein
MMP2	Matrix metallopeptidase 2
MT-ND5	Mitochondrially encoded NADH:Ubiquinone oxidoreductase core subunit 5
MYOC	Myocilin
NCAM1	Neural cell adhesion molecule 1
NEGR1	Neuronal growth regulator 1
NF1	Neurofibromin 1
NRXN1	Neurexin 1
p75 (NGFR)	Nerve growth factor receptor
PAX3	Paired Box 3
PCOLCE	Procollagen C-endopeptidase enhancer
PDGFRB	Platelet derived growth factor receptor beta
PECAM1	platelet and endothelial cell adhesion molecule 1
PTN	Pleiotrophin
PTPRZ1	Protein tyrosine phosphatase receptor type Z1
S100	S100 calcium binding protein A1
S100A12	S100 calcium binding protein A12
SCARB2	Scavenger receptor class B member 2
SERPINE2	Serpin family E member 2
SIRPA	Signal regulatory protein alpha
SLA-DRA	Swine leukocyte antigen DR alpha
SOX10	SRY-box transcription factor 10
SOX9	SRY-box transcription factor 9
SPARC	Secreted protein acidic and cysteine rich
SPON2	Spondin 2
SPP1	Secreted phosphoprotein 1
TAGLN	Transgelin
TGM3	Transglutaminase 3
THY1	Thy-1 cell surface antigen
TP53	Tumor Protein P53
VWF	von Willebrand factor
